# Effect of Temperature Gradient on the Grain Size Homogeneity of SEED Produced Semi-Solid Slurries by Phase-Field Simulation

**DOI:** 10.3390/ma12203309

**Published:** 2019-10-11

**Authors:** Wenying Qu, Min Luo, Zhipeng Guo, Xiaogang Hu, Ang Zhang, Fan Zhang, Daquan Li, Yongzhong Zhang

**Affiliations:** 1 General Research Institute for Nonferrous Metals, Beijing 101407, China; quwenying2008@163.com (W.Q.); lm_grinm@163.com (M.L.); lidaquan@grinm.com (D.L.); yyzhang@grinm.com (Y.Z.); 2 School of Materials Science and Engineering, Tsinghua University, Beijing 100084, China; zhipeng_guo@mail.tsinghua.edu.cn (Z.G.); za15@mails.tsinghua.edu.cn (A.Z.); 3 Department of Mechanical and Energy Engineering, Southern University of Science and Technology, Shenzhen 518055, China; huxg@sustech.edu.cn

**Keywords:** semi-solid, temperature gradient, grain size distribution, numerical visualization, phase-field

## Abstract

The distribution homogeneity of grain size affects the fluidity of the semi-solid slurry, which in turn affects the properties of the casting. One key factor affecting grain size uniformity resides in the nucleation number, which has been studied thoroughly, while the other factor is temperature gradient which has not been investigated yet. In this study, the microstructure evolutions under certain temperature gradients are investigated by experiment and simulation using a two-dimensional quantitative phase-field (PF) model. A parallel and adaptive mesh refinement algorithm is adopted to solve the nonlinear phase-field equations. The results indicate that temperature gradient can affect the size distribution of microstructure in the semi-solid slurry prepared by the SEED process. A higher temperature gradient (in the range of 0.230~0.657 °C/mm) along the radial direction is beneficial to the homogeneity of the grain size in a slurry.

## 1. Introduction

Semi-solid forming is proven to be a valid and high-efficiency solution to produce high integrity products for automotive industries compared with other forming processes [1]. Recently, the extension of this process in the type of alloys [2] and the size of products are important issues of concern, and the preparation of semi-solid slurries with uniform distribution of grain size is one of the key points. Several techniques have been invented for the generation of slurries with non-dendritic structure, such as cooling slope, low temperature pouring, vibration, gas bubbling, and swirling enthalpy equilibrium device (SEED) process [3]. The SEED process uses a liquid-based method (cooling from the temperature above liquidus) for producing a high-quality aluminium slurry that can be processed by high-pressure die casting. The principle of the SEED method is based on obtaining a rapid and controllable thermal equilibrium between a steel crucible and the bulk of molten aluminium to achieve slurries with uniformly distributed globular grains. These slurries have a high solid fraction and are free of entrapped oxides, allow for the casting of heat-treatable and weldable components with high mechanical properties, high ductility, and ultra-low porosity. It is high-efficient on a wide variety of aluminium foundry alloys and has been demonstrated on wrought alloys, such as 6061 [4] and 7075 alloys [5]. The weights and dimensions of slurries can be adjusted according to the die casting system, and the process can be applied in both horizontal and vertical die/squeeze casting machines. The important aspect of the semi-solid forming process is the quality of slurry, which determines the flow stability and the ultimate performance of products. Liu et al. [6] found that the morphology and uniformity of the primary phase have an important influence on the ultimate tensile strength and elongation of the casting. Understanding the effect of temperature gradient on microstructure formation is of great importance for the preparation of slurries with a more uniform microstructure, which is an urgent problem in the industrial application.

Computer technology has been well developed in materials science, it is a powerful tool to study troublesome physical problems based on fundamental physics, thermodynamics, kinetics, and so on. Provatas et al. [7] pointed out that solidification is the core of all forming techniques, and the simulation of microstructure evolution during the solidification process has been a subject of concern since a long time ago. Methods of stochastic, deterministic and others have been used to study this problem. Gawrońska et al. [8,9,10,11] investigated the effect of mechanical interactions between the casting and the mold on the conditions of heat dissipation which affects the solidification process and studied the solidification and the growth of dendrite by numerical simulation. While the fundamental study of semi-solid slurry microstructure evolution is very limited in literature, it is far behind the urgent need of an industrial application to understand the mechanism of typical semisolid microstructure formation. A mature and robust numerical model has not been established up to now. Only several researchers [12,13] have made some efforts to reveal the physical nature of the semi-solid structure formation in the cooling slope process or some particular problems such as the effect of melt flow pattern on the microstructure morphology by numerical method. 

The formation mechanism of microstructure under certain temperature gradient is always a focus in directional solidification. Zhang et al. [14] studied the microstructural evolution under a temperature gradient of 10 °C/mm by the CA model and compared with in-situ observation. Gandin et al. [15] studied the columnar-to-equiaxed transition (CET) of Al-7 wt.% Si alloys in directional solidification with a temperature gradient of 0.9 °C/mm, and found that the capillary-driven detachment during coarsening under microgravity is suggested as the potential mechanism for initiating the fragmentation. Beckermann et al. [16] modeled the freckle formation in single-crystal nickel-base superalloy directional solidification with a temperature gradient of 2~5 °C/mm and concluded that the onset of convection coincides with the occurrence of freckle defects and depends primarily on the primary dendrite arm spacing. Krane et al. [17] developed a cellular automaton-finite volume model for directional dendritic growth with a temperature gradient of 10 °C/mm. While, the temperature gradient existed in almost all of the solidification process, especially the melt solidification in a cylindrical mold. A study on the microstructural evolution along the radial direction can reflect the characteristics of the integrate microstructure. 

The phase-field (PF) method demonstrated by Kobayashi in 1994 [18] has also been developed extensively and rapidly in recent years. The PF method has an advantage in needless of explicit tracking of the solid-liquid interface during crystal growth compared with other methods. A variable φ is introduced in this method to represent the separation between solid and solid phases [19], −1 denotes liquid phase and 1 solid phase, values between −1 and 1 represents the interface of solid and liquid phases. The evolution of φ is a function of alloy internal and the interface gradient energies, then a set of partial differential equations (PDEs) are produced for fields of phase, solute and temperature. Due to the strongly coupled and non-linear characteristics of these PDEs, Gránásy et al. [20] adopted the simplified forms to study the crystal growth under isothermal condition, Lan et al. [21] studied a predefined and fixed temperature gradient. Karma et al. [19] developed a coupled solute and temperature PF model for dilute binary alloys, later on, Echebarria et al. [22] improved this model. A term named anti-trapping current, see Equation 1, was introduced into the solute conservation equation to eliminate the non-equilibrium effects.

(1) $${\vec{J}}_{a}=-\frac{W_{0}}{\sqrt{2}}\frac{C/C_{0}}{\left[1+k-\left(1-k\right)\varphi \right]}\frac{\partial \varphi }{\partial t}\frac{\nabla \varphi }{\left|\nabla \varphi \right|}$$

The term $W_{0}$ denotes the interface thickness, C and $C_{0}$ are the local and initial solute concentrations respectively, k is obtained from the binary alloy phase diagram, representing the equilibrium concentration partition coefficient, φ is the phase-field parameter. 

**Figure 1:**
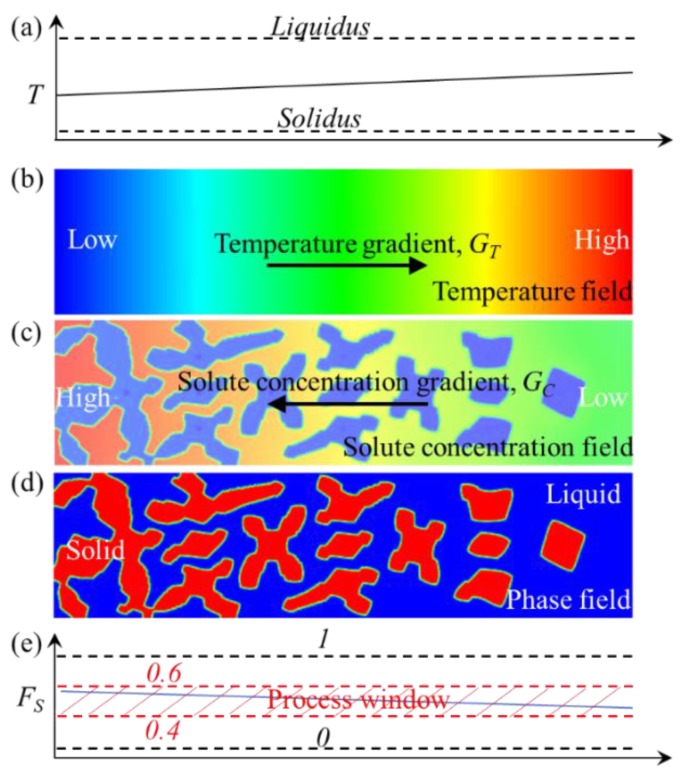
Schematic illustration of microstructure evolution under temperature gradient in mushy zones, (**a**) temperature distribution, (**b**) temperature field, (**c**) concentration distribution, (**d**) phase-field and (**e**) fraction solid and suitable process window of fraction solid for semi-solid die casting.

A rectangle 2D domain, see Figure 1, was selected as the Representative Effective Zone (REZ) to investigate the effect of temperature gradient on the distribution of grains. Under a certain temperature gradient, the local solute concentration differs due to the difference in growth speed of grain and diffuse coefficient caused by the driving force of various undercooling degrees. Then solid fraction will change along the radial direction, which should fall in the section of process window required by semi-solid die casting, as seen Figure 1e.

In this study, the microstructure evolution of a hypoeutectic Al-mass.7%Si binary alloy will be studied by using a 2D Phase-Field model. The characteristics and formation mechanism of the microstructures in semi-solid slurries will be discussed and proposals for preparation of slurries with a more uniform microstructure will also be investigated based on the numerical and experimental results. 

## 2. Experimental Approach

### 2.1. Materials and Properties

**Figure 2:**
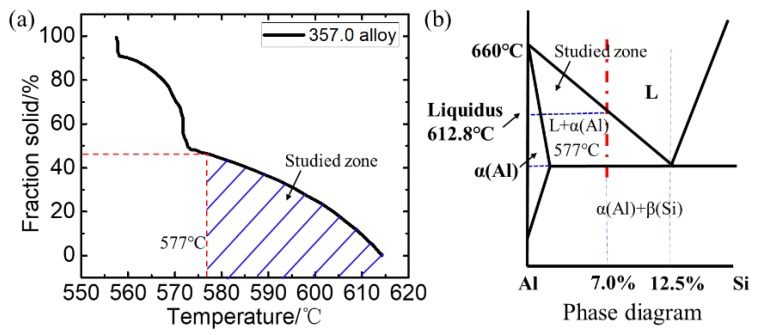
(**a**) Relationship between fraction solid and temperature, and (**b**) phase diagram for aluminum alloy 357.0.

The material used in this study is a commercial aluminium alloy 357.0 (supplied by Lizhong Alloy Group, Baoding, China). Its properties about the relationship between fraction solid and temperature (obtained by DSC test) can be seen in Figure 2a, the liquidus is 612.8 °C and the solidus is 557.2 °C. The phase diagram for Al-Si binary alloy is shown in Figure 2b. Above the eutectic temperature, only primary α-Al will precipitate from the bulk liquid. The thermodynamic and kinetic parameters of alloy 357.0 are listed in Table 1.

### 2.2. Slurry Preparation and Sample Observation

Semi-solid slurries are produced by the SEED process, where liquid alloy with a certain superheat is poured into a steel crucible and swirled until the desired semi-solid state is reached, then water quenching treatment for the whole semi-solid slurry is conducted. The swirling phase in the SEED process increases the forced convection, which can affect the distribution of temperature and solute concentration, thus the microstructure within the slurry. The experimental results about the microstructure made by the SEED process with and without swirling under pouring temperature of 630 °C indicate that the swirling phase has little influence on the microstructure. This is because the forced convection is weak in the slurry with high fraction solid made under low pouring temperature. This conclusion was also obtained from the effect of electromagnetic stirring on the microstructure of sand casting [23]. Then the swirling movement of the crucible was neglected in this study.

**Figure 3:**
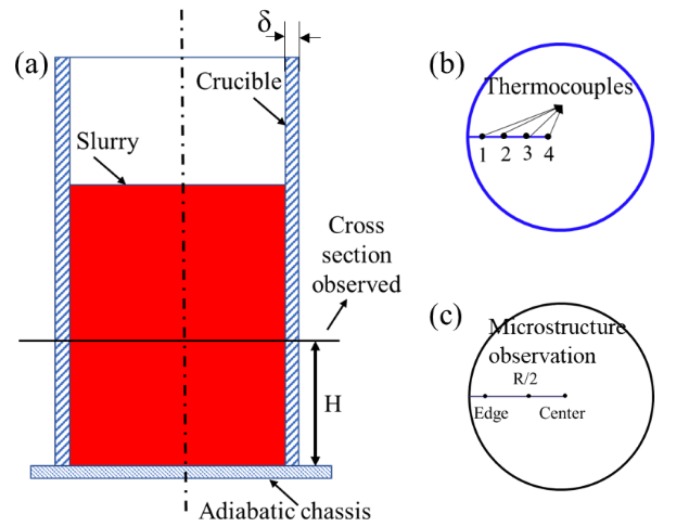
(**a**) Schematic of the crucible used in this study and the cross-sections for the crucible, H is the height of the observation plane, δ is the wall thickness of crucible, (**b**) temperature measuring points with thermocouples, (**c**) microstructure observation locations, named as Center, R/2 and Edge, respectively.

Three cooling conditions determined by the different wall thickness of crucibles were considered to study the radial temperature gradient effect on the difference of grain size. The schematics of the crucible, temperature measuring points, and locations for microstructure observation can be seen in Figure 3. The detail information about the crucible and process conditions is listed in Table 2. 

**Figure 4:**
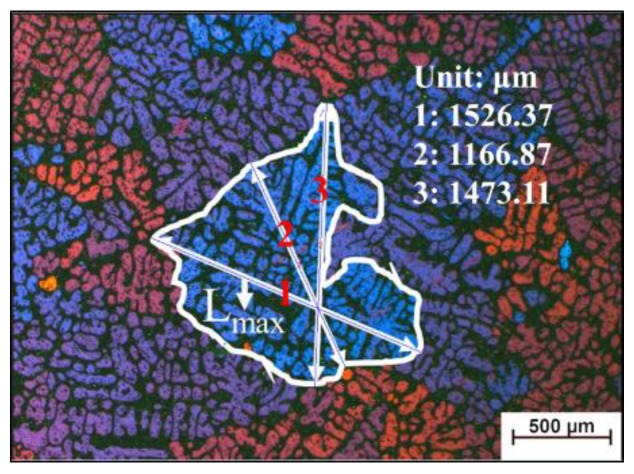
Schematic for the definition of grain size d, d = L_max_.

The temperature data was collected by a TOPRIE TP700 multipath data logger (Shenzhen toprank Electronics Co. Ltd., Shenzhen, China) with a sampling rate of 1 reading per second. The microstructures of these slurries after water quenching are grinded, polished, anodized with HBF4 reagent, then observed by optical microscope (OM, Chongqing Optec Instrument Co., Ltd, Chongqing, China) and polarized light microscopy (PLM, Carl Zeiss, Oberkochen, Germany). The grain size d is defined as the length of the longest axis of grain, as shown in Figure 4. Specific steps are as follows, determine the scale length, draw the outline of the grain, find its two longer axes, rotate around the intersection of the two axes to find the longest axis. All the statistical work of grain size in this article is done by using software Nano Measurer System 1.2 (Fudan University, Shanghai, China).

## 3. Model Description

The model employed in this article consists of two parts, a phase-field model for phase presentation, and the parallel computing and adaptive mesh refinement technique to improve calculation efficiency and precise description of the morphology. The code for FCC α-Al grain growth was written in Fortran 90. The phase-field model was developed based on the model explained by Ramirez and Beckermann [24,25] about the binary alloy solidification.

### 3.1. Phase-Field Model

The foundation theory used in the PF model is the Ginzburg-Landau Equation, see Equation (2), F is the total free energy of the study system, it has four variables, f is free energy density, C is solute concentration, φ represents phase, T is temperature, $k_{C}$, $k_{\varphi }$ and $k_{T}$ are concentration gradient energy coefficient of solute, phase gradient energy coefficient and temperature gradient energy coefficient, respectively.

(2) $$F\left(f,C,\varphi ,T\right)=\underset{V}{\int }[f\left(C,\varphi ,T\right)+\frac{k_{c}}{2}{\left(\nabla C\right)}^{2}+\frac{k_{\varphi }}{2}{\left(\nabla \varphi \right)}^{2}+\frac{k_{T}}{2}{\left(\nabla T\right)}^{2}]dv$$

The governing equations incorporating convection are as follows [26]:

(3) $$\tau_{0}\frac{\partial \varphi }{\partial t}=-k_{\varphi }\frac{\delta F}{\delta \varphi }$$

(4) $$\frac{\partial C}{\partial t}+f_{l}\times \nabla C=\nabla \times \left(k_{C}\nabla \frac{\delta F}{\delta C}-{\vec{J}}_{a}\right)$$

In Equation (4), $f_{l}$ is the liquid fraction, $f_{l}$ = (1−φ)/2, the term ${\vec{J}}_{a}$ is used to solve the solute trapping problem at the solid-liquid interface, it was provided by Karma and co-workers in 2001 [19]. 

The laws described by the model should be independent of the effect of dimensionality, and further discussion of the mechanism model should exclude the effect of dimensionality. So, first the terms of solute concentration c and temperature T are transformed into dimensionless forms U and θ, respectively, see Equations (5) and (6).

(5) $$U=\frac{\frac{2C/C_{0}}{1+k-\left(1-k\right)\varphi }-1}{1-k}$$

(6) $$\theta =\frac{T-T_{M}-mC_{0}}{\Delta T_{0}}$$

where $T_{M}$ is the melting temperature of the solvent, m is the liquidus slope, $\Delta T_{0}=\left|m\right|C_{0}\left(1-k\right)/k$ is the equilibrium freezing temperature range. The dimensionless forms of time and length scales can be seen in Equations (7) and (8).

(7) $$\tau_{0}=d_{0}^{2}a_{2}\lambda^{3}/\left(Da_{1}^{2}\right)$$

(8) $$W_{0}=\lambda d_{0}/a_{1}$$

The term $\tau_{0}$ is the relaxation time, $d_{0}$ is the chemical capillary length, $d_{0}=\Gamma /\Delta T_{0}$, Γ is the Gibbs-Thomson coefficient, $D=\lambda a_{2}$ is the dimensionless solute diffusivity. Parameter λ is the coupling constant defined as:

(9) $$\lambda =\frac{15RT_{M}\left(1-k\right)}{16v_{0}h\left|m\right|}\Delta T_{0}$$

The term R is the gas constant, $v_{0}$ is the molar volume, and h is the energy barrier of the double-well potential. And $a_{1}=$ 0.8839, $a_{2}=$ 0.6267 [24].

Then the governing equations for phase-field and solute field are expressed as:

(10) $$\begin{array}{c} A{\left(\psi \right)}^{2}\left[MC_{0}\left(1+\left(1-k\right)U\right)\right]\frac{\partial \varphi }{\partial t}\\ \;\;\;\;\;\;\;=\nabla \times \left(A{\left(\psi \right)}^{2}\nabla \varphi \right)-\frac{\partial }{\partial x}\left(A\left(\psi \right)A{\left(\psi \right)}^{\prime }\frac{\partial \varphi }{\partial y}\right)+\frac{\partial }{\partial y}\left(A\left(\psi \right)A{\left(\psi \right)}^{\prime }\frac{\partial \varphi }{\partial x}\right)\\ \;\;\;\;\;\;\;+\varphi \left(1-\varphi^{2}\right)-\lambda {\left(1-\varphi^{2}\right)}^{2}\left(\theta +MC_{0}U\right) \end{array}$$

and:

(11) $$\begin{array}{c} \left(\frac{1+k}{2}-\frac{1-k}{2}\varphi \right)\frac{\partial U}{\partial t}\\ \;\;\;\;\;=\nabla \times \left(D\frac{1-\varphi }{2}\nabla U+\frac{1}{2\sqrt{2}}\left[1+\left(1-k\right)U\right]\frac{\partial \varphi }{\partial t}\frac{\nabla \varphi }{\left|\nabla \varphi \right|}\right)\\ \;\;\;\;\;+\frac{1}{2}\left[1+\left(1-k\right)U\right]\frac{\partial \varphi }{\partial t}-\frac{1}{2}f_{l}\times \left\{\left[1+k-\left(1-k\right)\varphi \right]\nabla U-[1+\left(1-k\right)U]\nabla \varphi \right\} \end{array}$$

In Equation (10), $A\left(\psi \right)=1+\varepsilon \;\cos\left[4\left(\psi -\psi_{0}\right)\right]$ is the anisotropy function, ε denotes the anisotropy strength, $\psi =\arctan\left(\varphi_{x}/\varphi_{y}\right)$ is the angle between the primary arm and x-axis, $\varphi_{x}=\partial \varphi /\partial x$, $\varphi_{y}=\partial \varphi /\partial y$, and $\psi_{0}$ is the predefined growth orientation. $M=\left|m\right|\left(1-k\right)/\Delta T_{0}$, is dimensionless liquidus slope.

### 3.2. Parallel Computing and Adaptive Mesh Refinement

**Figure 5:**
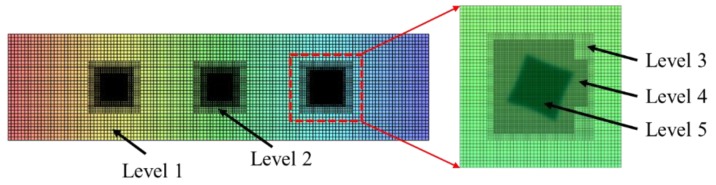
Schematic for the mesh created for computing domain by using the adaptive mesh refinement (AMR) technique.

A robust algorithm [27,28] combining the parallel computing and adaptive mesh refinement (AMR) which effect can be seen in Figure 5, is employed to improve the computing efficiency in this article. The parallel computing was realized by multi-core architectures based on MPI (message-passing interface). The governing equations about phase and solute fields (Equations (10) and (11)) were discretized using the finite difference method onto a rectangle computing domain with an equal grid spacing of Δx=Δy and sloved by the robust algorithm. The details about space and time discretization, and parallel computing scheme can be found in reference [28,29]. In this article, a server (Dell, Inc., Roderock, TX, USA) with 416 processors in GRINM (General Research Institute for Nonferrous Metals) was used to solve the cases designed according to the experimental data. 

## 4. Results and Discussion

### 4.1. Thermal Conditions and Corresponding Microstructures in Slurries

**Figure 6:**
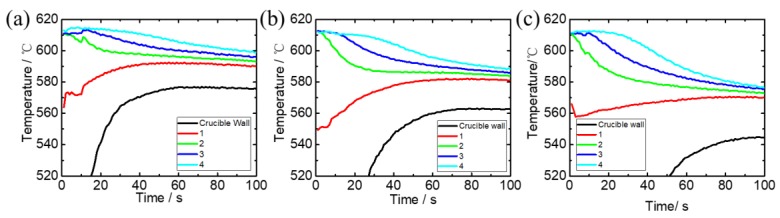
Temperature measuring results for the three slurries in crucibles with different wall thickness, (**a**) 2.8 mm, (**b**) 4.2 mm, (**c**) 5.6 mm.

The temperature measuring results for the three crucibles with different wall thickness can be seen in Figure 6. For the temperature of point 1 is significantly influenced by the wall and it changes dramatically, then the temperature gradient is determined by points 2~4 (the distance between points 2 and 4 is 30 mm) during the first 20 s. Temperature gradients for the three cases are set as 0.230 °C/mm, 0.442 °C/mm, 0.657 °C/mm, respectively.

**Figure 7:**
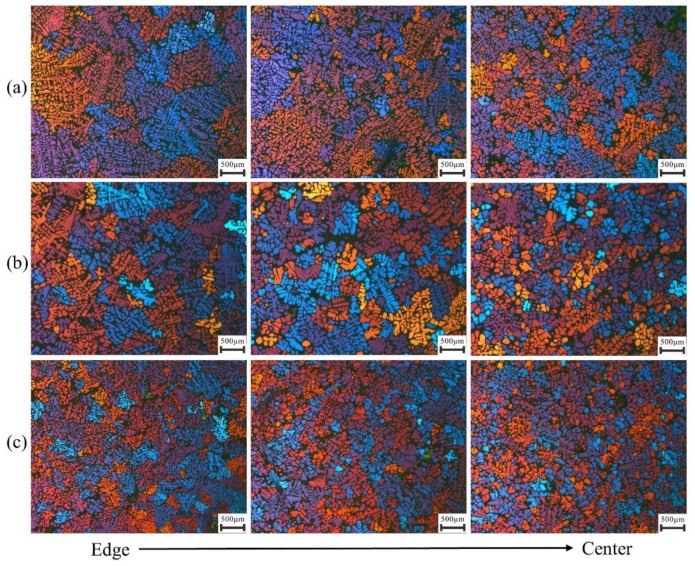
Experimental results about the microstructures from edge to center in slurries made by crucibles with different temperature gradients, (**a**) 0.230 °C/mm, (**b**) 0.442 °C/mm and (**c**) 0.657 °C/mm.

The corresponding microstructures made by crucibles with different wall thickness can be seen in Figure 7. From the microstructures at the three locations along the radial direction in the slurries can see that the difference between grain size at the center and edge side is decreasing with the increase of the wall thickness, i.e. the increase of temperature gradient. 

The nucleation number will increase sharply due to the increasing chill effect of the crucible wall, the role of temperature gradient, which is almost covered by the nucleation effect should not be neglected. 

### 4.2. Numerical Simulation Results

Based on the above thermal conditions for the microstructure formation, numerical cases were designed to investigate the evolution mechanism of microstructure under different temperature gradients. The temperature variation along the radial direction was assumed as constant, and the temperature gradient is properly set based on the experimental results. The detailed information of the simulation conditions can be seen in Table 3. The domain for simulation was set based on the upscaled method, which means the small REZ can reflect the phenomenon that happened on a large scale. Although the domains are small compared with the actual condition, it can give the variation trend of the dependent variable with the change of parameters, reveal the internal mechanism. The results were withdrawn at the simulation time of 100,000 Δt and 300,000 Δt.

Major assumptions should be made as follows, the temperature at the center is set as 612.8 °C for the three cases. There is no cooling effect and no melt convection, the effect of nucleation of this process was neglected. The same number of nuclei was assumed and these nuclei have the same initial diameter. The latent heat during solidification was also not considered. 

**Figure 8:**
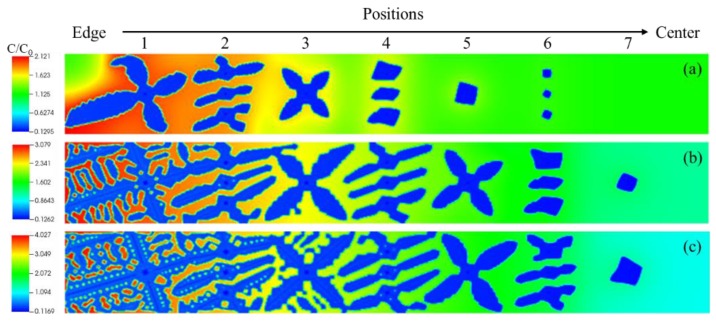
Distribution of solute concentration at time step 100,000 Δt under different temperature gradients, (**a**) 0.230 °C/mm, (**b**) 0.442 °C/mm and (**c**) 0.657 °C/mm.

The simulation results for the three cases can be seen in Figure 8. As the temperature gradient increases, the uniformity of microstructure becomes better. Under the highest temperature gradient of 0.657 °C/mm, the secondary arms are already detached away from the primary dendrite. When the grain at the edge side is almost fully grown up, the one at the center has not started to grow and even disappeared in the case of the lowest temperature gradient of 0.230 °C/mm.

This characteristic of microstructure distribution can coincide well with the experimental results in Figure 7, with the increase of temperature gradient, the degree of homogeneity is increasing. The coincidence between experimental result and simulation indicates that the temperature gradient along radial direction has a significant influence on the microstructure and the size of REZ is acceptable. 

**Figure 9:**
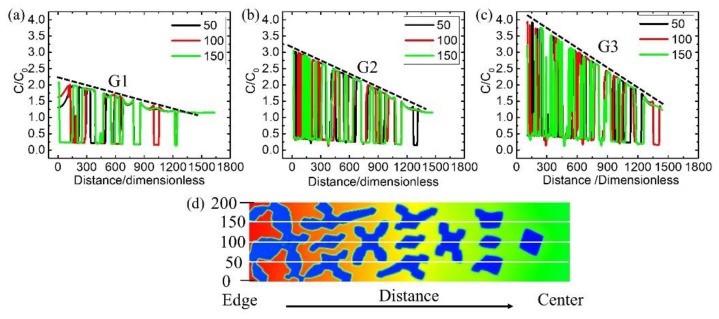
Simulation results about the solute concentration distribution under different temperature gradient (**a**) 0.230 °C/mm, (**b**) 0.442 °C/mm and (**c**) 0.657 °C/mm along the three white lines in (**d**).

To investigate the reason for the difference between these microstructures, the distribution of solute concentration in the REZ was analyzed by extracting data from the three parallel lines defined in Figure 9d. Figure 9a–c demonstrates that the concentration gradient of solute increases with the increase of temperature gradient G in the slurry, G1 < G2 < G3 and the gradient is in the opposite direction compared with that of temperature. The concentration of solute at the edge side can result in the decrease of local liquidus, then the undercooling degree is reduced, the driving force for grain growth is becoming smaller. The concentration of solute around the solid-liquid interface can hinder the growth of the primary dendrite and may remelt the secondary arms. Then, the morphology of the dendrite will change significantly.

The simulation and experimental results indicate that the temperature gradient can affect the distribution of grain size, under high-temperature gradient the distribution is more uniform. 

**Figure 10:**
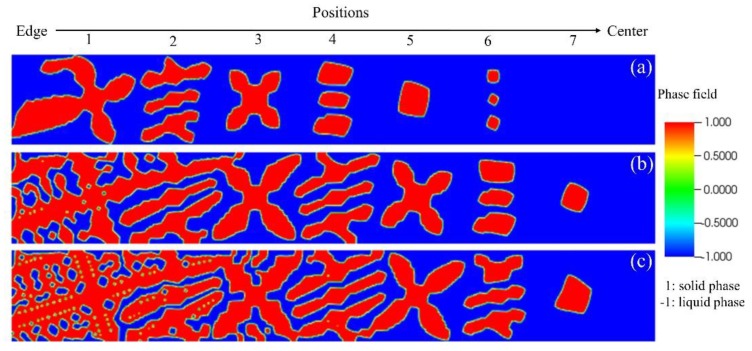
Simulation results about the phase-field of the three conditions with different temperature gradients at time step 300,000 Δt, (**a**) 0.230 °C/mm, (**b**) 0.442 °C/mm and (**c**) 0.657 °C/mm.

To confirm the evolution of microstructure with time, simulation results at time step 300,000 Δt were also examined, see Figure 10. The results indicate that the bigger temperature gradient can reduce the size difference of grains at edge and center along the radial direction gradually. At the edge side, the secondary arms are also detached away from the primary dendrite under the temperature gradient of 0.442 °C/mm. While the lower temperature gradient will increase the size difference of grains with time due to the difference in growth speed. 

### 4.3. Discussion

Due to the directivity of heat transfer, temperature gradient is inevitable during solidification, and temperature gradient can affect the microstructure. The temperature gradient in directional solidification can influence the melting of secondary arms which is essential for dendrite arm migration, and the typical value is about 12 °C/mm [14]. While, in semi-solid slurry preparation, this value is about 0.2~1.0 °C/mm. 

**Figure 11:**
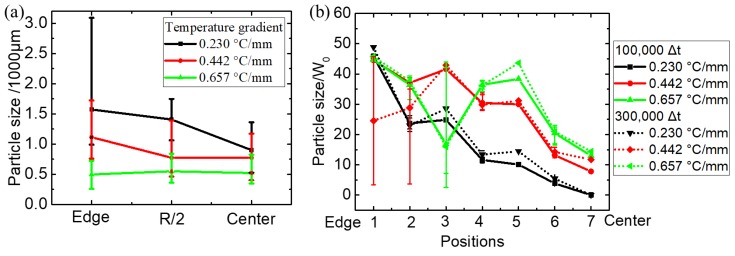
Statistical results about grain size of (**a**) experiment and (**b**) simulation.

The statistical results about experiment and simulation can be seen in Figure 11. Figure 11a shows that the difference in grain size from edge to center decreases with the increase of temperature gradient in the experiment. The simulation results shown in Figure 11b present the same trend as the experimental results, indicating that a higher temperature gradient is conducive to reducing the grain size difference between the edge and the center of the slurry.

It should also be noted that the overall grain size in the simulation results increases with the increase of temperature gradient, which is inconsistent with the experimental results. The reason is that the difference in nucleation rate under different cooling conditions is not considered in this paper. The higher the temperature gradient, the stronger the cooling ability, the higher the nucleation number and the smaller the grain size. The effect of pouring temperature on grain size and distribution uniformity of microstructure was studied by Liu et al. [6] and Liang et al. [30]. They found that the lower the pouring temperature, the stronger the cooling ability of the crucible to the slurry, and the more uniform and finer the microstructure. Compared with the previous studies, it can be found that the change of nucleation number caused by cooling ability is a significant factor affecting the uniformity and size of microstructure, while the internal temperature gradient is a parameter neglected. Conventional experimental studies cannot consider the effects of nucleation and temperature gradient on microstructure separately. However, the simulation is able to study the effect of temperature gradient on microstructure separately on the basis of certain assumptions. This is also the purpose of this paper. Although the influence of temperature gradient on microstructure is studied in this paper, the cross-influence of nucleation effect and temperature gradient on microstructure cannot be obtained, which will be further investigated in the next step.

The variation of grain size along the radial direction is determined by distributions of temperature and solute concentration within the slurry. The temperature is controlled by the crucible which initial temperature is 25 °C. The thermal parameters are assumed to be constant and the corresponding values of the initial state are used. The interface heat transfer coefficient h between slurry and crucible is assumed to be 1000 W·m^−2^·K^−1^, the thermal conductivity λ of the steel crucible is about 21.488 W·m^−1^·K^−1^. The wall thicknesses δ are 2.8, 4.2, 5.6 mm, respectively. Then the Biot number (Bi = δh/λ) for the three cases is 0.130, 0.196 and 0.261, respectively, which means the thermal gradient within the crucibles is small and almost the same. It indicates that the surface temperature of the crucible (the black line in cooling curves) can represent the temperature of the inner surface of the crucible.

The black line in Figure 6 is the temperature variation of the crucible wall. The temperature difference between the crucible wall and slurry is increasing with the crucible thickness. The wall temperature affects the temperature distribution inside the slurry, a lower wall temperature producing a higher temperature gradient in the slurry. The temperature near-surface (line 1 in Figure 6) is increasing with time, then the driving force for grain growth is reducing. Comparing the three cases, it may be doubtful that the temperature rise near-surface is greater in the case with a lower temperature gradient but there is no obvious dendrite arm fracture in the simulation. This phenomenon can be explained as follows, the growth rate of grain under low temperature gradient is slow and the secondary dendrite arm is stronger, the fracture of dendrite becomes difficult.

**Figure 12:**
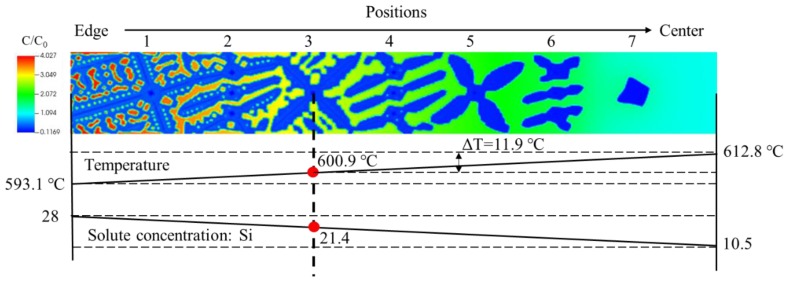
Schematic of the conditions about temperature and solute concentration for detachment of secondary arms.

From the results obtained above, it can be seen that the increase of temperature can increase the solute concentration gradient, the higher the solute concentration at the edge side, the stronger the inhibition of dendrite growth, and when the solute is enriched to a certain degree, secondary arms will detach away from the main body. According to the distributions of temperature and solute concentration within the slurry from simulation result, take the case with a temperature gradient of 0.657 °C/mm at 100,000 Δt for example, the undercooling degree for detachment of secondary arms due to solute enrichment is estimated. The results can be seen in Figure 12. The phenomenon of detachment can occur when the undercooling degree is reached 11.9 °C.

**Figure 13:**
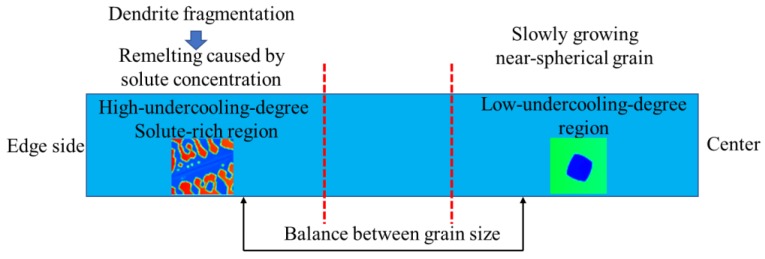
The balance between grain size under different local conditions from edge side to center.

The uniform evolution of grain size needs a balance between space and time, space is determined by local solidification speed, solute concentration, and actual undercooling degree, the time is dominated by the competition among driving force in the active region. Schematic about the balance between edge side and center about the grain size can be seen in Figure 13. The grain at the center will grow slowly under a low undercooling degree. Although the grain at the edge side can grow fast due to the high undercooling degree, its secondary arms will remelt from the bottom for the solute concentration. Then the difference of grain size between edge and center is gradually reduced in dynamic adjustment.

## 5. Conclusions

This study has investigated the effect of radial temperature gradient on the grain size distribution in semi-solid slurries by using the phase-field method. With the increase of temperature gradient, the gradient of solute concentration is increasing. The hinder effect on grain growth and detachment effect on secondary arms from solute enrichment at edge side can decrease the grain size, then reduce the difference of grain size between the edge side and center. A higher temperature gradient (in the range of 0.230~0.657 °C/mm) along the radial direction is beneficial to the uniformity of the grain size in a slurry prepared by the SEED process. However, one thing to note is that, if the temperature gradient is too high, it will produce a solid shell adjacent to the crucible wall, which is not good for die filling. 

## Figures and Tables

**Table 1:** Thermodynamic and kinetic parameters of aluminium alloy 357.0.

Symbol	Definition	Unit	Value
*k*	Solute partition coefficient	/	0.1147
*m_L_*	Liquidus slope	°C·wt.%^−1^	−6.79
*Γ*	Gibbs-Thomson coefficient	/	2 × 10^−7^
*C_P_*	Specific heat of the liquid	J·kg^−1^·°C^−1^	963
*D_L_*	Diffusion coefficient for Si in liquid	m^2^·s^-1^	3 × 10^−9^
*σ*	Interfacial energy	J·m^−2^	0.2
*M_Φ_*	Phase-field mobility	m^3^·J^−1^·s^−1^	0.34
$c_{\infty }$	Initial solute concentration	/	0.07
$w_{0}$	Initial composition of alloy	/	0.07
$w_{e}$	Eutectic composition of silicon in liquid	/	0.126
$w_{es}$	Eutectic composition of silicon in α-Al	/	0.0148

**Table 2:** The detail information about the crucible and process conditions.

Crucible Diameter/mm	Wall Thickness δ/mm	Slug Weight/kg	Pouring Temperature/°C	Height of Observation Plane/mm	Crucible Temperature/°C
92	2.8	2.385 ± 0.215	630	90	25
	4.2				
	5.6				

**Table 3:** Simulation parameters for the microstructure evolution under different conditions.

Cases	Domain/*W*_0_	Temperature Field	Nuclei Number	Temperature Gradient/°C/mm	Result Acquisition Time/Δt (Simulation Time Steps)
a	32 × 226	Left-right corresponding to Low-High	13	0.230	100,000, 300,000
b				0.442	
c				0.657	
